# Multi-objective dynamic population shuffled frog-leaping biclustering of microarray data

**DOI:** 10.1186/1471-2164-13-S3-S6

**Published:** 2012-06-11

**Authors:** Junwan Liu, Zhoujun Li, Xiaohua Hu, Yiming Chen, Feifei Liu

**Affiliations:** 1 School of Computer and Information Engineering, Central South University of Forestry and Technology, Changsha 410004, China; 2 State Key Laboratory of Software Development Environment, Beihang University, Beijing 100191, China; 3 Beijing Key Laboratory of Network Technology, Beihang University, Beijing 100191, China; 4 Department of Computer Science, Central China Normal University, Wuhan 430079, China; 5 College of Information Science, Drexel University, Philadelphia, PA 19104, USA; 6 School of Information Science and Technology, Hunan Agricultural University, Changsha 410128, China; 7 Library, Central South University of Forestry and Technology, Changsha 410004, China

## Abstract

**Background:**

Multi-objective optimization (MOO) involves optimization problems with multiple objectives. Generally, theose objectives is used to estimate very different aspects of the solutions, and these aspects are often in conflict with each other. MOO first gets a Pareto set, and then looks for both commonality and systematic variations across the set. For the large-scale data sets, heuristic search algorithms such as EA combined with MOO techniques are ideal. Newly DNA microarray technology may study the transcriptional response of a complete genome to different experimental conditions and yield a lot of large-scale datasets. Biclustering technique can simultaneously cluster rows and columns of a dataset, and hlep to extract more accurate information from those datasets. Biclustering need optimize several conflicting objectives, and can be solved with MOO methods. As a heuristics-based optimization approach, the particle swarm optimization (PSO) simulate the movements of a bird flock finding food. The shuffled frog-leaping algorithm (SFL) is a population-based cooperative search metaphor combining the benefits of the local search of PSO and the global shuffled of information of the complex evolution technique. SFL is used to solve the optimization problems of the large-scale datasets.

**Results:**

This paper integrates dynamic population strategy and shuffled frog-leaping algorithm into biclustering of microarray data, and proposes a novel multi-objective dynamic population shuffled frog-leaping biclustering (MODPSFLB) algorithm to mine maximum bicluesters from microarray data. Experimental results show that the proposed MODPSFLB algorithm can effectively find significant biological structures in terms of related biological processes, components and molecular functions.

**Conclusions:**

The proposed MODPSFLB algorithm has good diversity and fast convergence of Pareto solutions and will become a powerful systematic functional analysis in genome research.

## Background

With rapid development of the DNA microarray technology, simultaneously measuring the expression levels of thousands of genes in a single experiment can yield large-scale datasets. The analysis of microarray data mainly contains the study of gene expression under different environmental stress conditions and the comparisons of gene expression profiles for tumors from cancer patients. A subset of genes showing correlated co-expression patterns across a subset of conditions are expected to be functionally related. By comparing gene expression in normal and disease sells, microarray dataset may be used to identify disease genes and targets for therapeutic drugs. Therefore, mining patterns from microarray dataset becomes more and more important. These patterns relate to disease diagnosis, drug discovery, protein network analysis, gene regulate, as well as function prediction.

For microarray data analysis, clustering techniques is a popular technique for mining significant biological models. Clustering can identify set of genes with similar profiles. However, traditional clustering approaches such as k-means [1], self organizing maps [2], support vector machine [3] and hierarchical clustering [4], assume that related genes have the similar expression patterns across all conditions, which is not reasonable especially when the dataset contains many heterogeneous conditions. It fact, those relevant genes are not necessarily related to all conditions. To cluster subset of genes that have similar expression over some conditions, biclustering [5,6] is proposed for clustering simultaneously gene subset and condition subset over which the gene subset exhibit similar expression patterns, such as δ-biclustering [5], pClustering [7], statistical-algorithmic method for biclustering analysis (SAMBA) [8], spectral biclustering [9], Gibbs sampling biclustering [10] and simulated annealing biclustering [11].

In recent three decades, inspired by biology views, heuristics optimization has become a very popular research topic. To order to escape from local minima, many evolutionary algorithms (EA) are used to find global optimal solutions from gene expression data [12-14]. If a single objective is optimized, the global optimum solution can be found. But in the real-world optimization problem, there are several objectives in conflict with each other to be optimized and require different mathematical and algorithmic tools to solve it. Multi-objective evolutionary algorithm (MOEA) generates a set of Pareto-optimal solutions [15] which is suitable to optimize two or more conflicting objectives.

However when mining biclusters from microarray data, we must optimize simultaneously several objectives in conflict with each other, for example, the size and the homogeneity of the clusters. In this case MOEA is proposed to discover efficiently global optimal solution. Among many MOEA proposed, the relaxed forms of Pareto dominance has become a popular mechanism to regulate convergence of an MOEA, to encourage more exploration and to provide more diversity. Among these mechanisms, ϵ-dominance has become increasingly popular [16], because of its effectiveness and its sound theoretical foundation. ϵ-dominance can control the granularity of the approximation of the Pareto front obtained to accelerate convergence and guarantee optimal distribution of solutions. At present, several algorithms [17,18] adopt MOEAs to discover biclusters from microarray data.

Recently particle swarm optimization (PSO) proposed by Kebnnedy and Eberhart [19] is a heuristics-based optimization approach simulating the movements of a bird flock finding food. Most of previous versions of the particle swarm are based on continuous space, where trajectories are the changes of position on some dimensions. Kennedy and Eberhart [20] proposed a discrete binary version of PSO, where trajectories are defined as changes of probability that a coordinate will take on a zero or one value.

The most attractive of PSO is that there are very few parameters to adjust. So it has been successfully used for both continuous nonlinear and discrete binary single-objective optimization.

The rapid convergence and relative simplicity of PSO make it very suitable to solve multi-objective optimization named as multi-objective PSO (MOPSO). In recent years many multi-objective PSO (MOPSO) approaches [21,22] has proposed. The strategy of ϵ-dominance [23,24] is introduced into MOPSO speeding up the convergence and attaining good diversity of solutions [25]. Liu [26] incorporates ϵ-dominance strategies into MOPSO, and proposes a novel MOPSO biclustering framework to find one or more significant biclusters of maximum size from microarray data.

Most MOPs use a fixed population size to find non-dominated solutions for obtaining the Paterto front. The computational cost is the greatest influence of population size on these population-based meta-heuristic algorithms. Hence dynamically adjusting the population size need consider the balance between computational cost and the algorithm performance. Some methods using dynamic size are proposed. Tan [27] proposed an incrementing MOEA (IMOEA) that adaptively computes am appropriate population size according to the online discovered trade-off surface and its desired population size that corresponds to the distribution density. Yen and Lu [28] proposed dynamic population size MOEA (DMOEA) that includes a population-growing strategy based on the converted fitness and a population-declining strategy that resorts to the following age, health and crowdedness. Leong and Yen [29] introduced dynamic population size and a fixed number of multiple swarms into multi-objective optimization algorithm that improved diversity and convergence of optimization algorithm. Based on dynamic population, Liu [30] proposed a novel dynamic multi-objective particle swarm optimization biclustering (DMOPSOB) algorithm to mine effectively significant biclusters of high quality.

In recent years, Eusuff [31,32] develops a shuffled frog-leaping algorithm (SFLA) to solve combinatorial optimization problems. Due to its effectiveness and suitability, SFLA has captured much attention and been applied to solve many practical optimization problems [31-33]. The shuffled frog leaping (SFL) optimization algorithm has been successful in solving a wide range of real-valued optimization problems. Madani [34] proposes a discrete shuffled particle optimization algorithm with best performance in terms of both success rate and speed than the binary genetic algorithm (BGA) and the discrete particle swarm optimization (DPSO) algorithm.

To the best of our knowledge, there is no published work dealing with the biclustering of microarray data by using SFLA. Thus, in this paper we present an effective SFLA biclustering algorithm for mining the maximum biclusters with allowable dissimilarity within the biclusters, and with a greater row variance. Computational experiments and comparisons show that the proposed SFLA outperforms three best performing algorithms proposed recently for solving the biclustering problem with the biclustering criterion.

## Methods

Based on shuffled frog-leaping algorithm, crowding distance and ε-dominance strategy [16], this paper incorporating dynamic population strategy into MOSFLB algorithm [35], and proposes a multi-objective dynamic population shuffled frog-leaping biclustering (MODPSFLB) algorithm to mine one or more significant biclusters of maximum size from microarray dataset. In the proposed algorithm, the feasible solutions are regarded as frogs and Pareto optimal solutions are preserved in frog population updated by ε-dominance relation and computation of crowding distance. Then the next generation of frog population is dynamically adjusted according to dynamic population strategy [29]. The proposed methods can effectively obtain more Pareto optimal solutions that uniformly distributed onto the Pareto front. The proposed algorithm uses three objectives, the size, homogeneity and row variance of biclusters, as three fitness function of biclustering optimization process. A low mean squared residue (MSR) score of bicluster denotes that the expression level of each gene within the bicluster is similar over the range of conditions. Therefore, the goal of the algorithm is to find more maximum biclusters with mean squared residue lower than a given δ and with a relatively high row variance.

### Biclusters

Given a gene expression data matrix D = G×C = (here *i*∈[1, *n*], *j*∈[1, m]) is a real-valued *n*×*m *matrix, here G is a set of n genes {g_1_, g_2_,..., g_n_}, C a set of m biological conditions {c_1_, c_2_,..., c_n_}. Entry *d_ij _*means the expression level of gene *g_i _*under condition c*_j_*.

**Definition 1 Bicluster**. Given a gene expression dataset D = G×C, if there is a submatrix B = g×c, where g⊂G, c⊂C, to satisfy certain homogeneity and minimal size of the cluster, we say that B is a bicluster.

**Definition 2 Maximal bicluster**. A bicluster B = g×c is maximal if there exists not any other biclusters B'B'= g'×c' g'×c' such that, g'⊂g, c'⊂C.

**Definition 3 Dimension mean**. Given a bicluster B = g×c, with subset of genes g⊂G, subset of conditions c⊂C, *d_ij _*is the value of gene *g_i _*under condition c*_j _*in the dataset D. We denote by d_ic_d_ic _the mean of the ith gene in B, d_gj _the mean of the jth condition in B. We also denote by d_gc _the mean of all entries in B. These values are defined as follows, where Size(g, c) = |g||c| presents the size of bicluster B.

(1) $$d_{ic}=\frac{1}{\left|c\right|}\sum_{j\in c}d_{ij}$$

(2) $$d_{gj}=\frac{1}{\left|g\right|}\sum_{i\in g}d_{ij}$$

(3) $$d_{gc}=\frac{1}{\left|g||c\right|}\sum_{i\in g,j\in c}d_{ij}$$

**Definition 4 Residue and mean square residue**. Given a bicluster B = g×c, to assess the difference the actual value of an element *d_ij _*and its expected value, we define by r(d_ij_) the residue of d_ij _in bicluster B in Eq.(4). Therefore the mean squared residue (MSR) of B is defined as the sum of the squared residues to assess overall quality of a bicluster B in Eq.(5).

(4) $$r\left(d_{ij}\right)=d_{ij}-d_{ic}-d_{gj}+d_{gc}$$

(5) $$MSR\left(g,c\right)=\frac{1}{\left|g||c\right|}\sum_{i\in g,j\in c}r{\left(d_{ij}\right)}^{2}$$

**Definition 5 Row variance**. Given a bicluster B = g×c, the ith gene variance in B is defined by RVAR(i, c) and the overall gene-dimensional variance is defined as the sum of all genes variance as follows.

(6) $$RVAR\left(g,c\right)=\frac{1}{\left|g||c\right|}\sum_{i\in g,j\in c}{\left(d_{ij}-d_{ic}\right)}^{2}$$

(7) $$RVAR\left(i,c\right)=\frac{1}{\left|c\right|}\sum_{j\in c}{\left(d_{ij}-d_{ic}\right)}^{2}$$

Our target is mining good quality biclusters of maximum size, with mean square residue (MSR) smaller than a user-defined threshold **δ **> 0, which presents the maximum allowable dissimilarity within the biclusters, and with a greater row variance. The problem is NP-complete, so the large majority of the algorithms use heuristic approaches to attain near optimal solutions.

### Bicluster encoding

**Figure 1:**
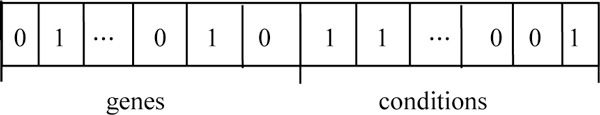
**An individual encoding a bicluster**. Figure 1 presents the individual encoding a bicluster with 2 genes and 3 conditions, and its size is 2 × 3 = 6.

Each bicluster is encoded as an individual of the population. Each individual is represented by a binary string of fixed length *n*+*m*, where *n, m *is the number of genes, conditions of the microarray dataset, respectively. The first n bits are responding to n genes, the following m bits to m conditions. If a bit is set to 1, it means that the responding gene or condition belongs to the encoded bicluster; otherwise it does not. This encoding method presents the advantage of having a fixed size, thus using standard variation operations. Figure 1 presents the individual encoding a bicluster with 2 genes and 3 conditions, and its size is 2 × 3 = 6.

### Fitness function

We hope to mine those biclusters with low mean squared residue, with high volume and gene-dimensional variance, thus three objectives in conflict with each other are used to model multi-objective optimization problem. In this paper, we use the following three fitness functions [26].

(8) $$f_{1}\left(x\right)=\frac{\left|G||C\right|}{size\left(x\right)}$$

(9) $$f_{2}\left(x\right)=\frac{MSR\left(x\right)}{\delta }$$

(10) $$f_{3}\left(x\right)=\frac{1}{RVAR\left(x\right)}$$

Where G and C are the total number of genes and conditions of the microarray datasets respectively. Size(x), MSR(x) and RVAR(x) denotes the size, mean squared residue and row variance of bicluster encoded by the frog × respectively. δ is the user-defined threshold for the maximum acceptable mean squared residue. Our algorithm minimizes those three fitness functions.

### ϵ-dominance

Among many MOEA proposed, the non-dominated solutions of each generation are kept in an external population that must be updated in each generation. The time needed for updating the population depends on the population size, population size and the number of objectives and increases extremely when increasing the values of these three factors [36]. To encourage more exploration and to provide more diversity the relaxed forms of Pareto dominance has become a popular mechanism to regulate convergence of an MOEA. Among these mechanisms, ϵ-dominance has become increasingly popular [16], because of its effectiveness and its sound theoretical foundation. ϵ-dominance can control the granularity of the approximation of the Pareto front obtained to accelerate convergence and guarantee optimal distribution of solutions. Here, we adapt the idea of ϵ-dominance to fix the size of the population to a certain amount. This size depends on ϵ. We apply ϵ-dominance technique to search for the approximate Pareto-front.

**Definition 6 Dominance relation**. Let f, g ∈R^m^. Then f is said to dominate g (denoted as f ≻ g), iff

**(i) **∀i ∈**{1,...., m}**: f_i _≤ g_i_

**(ii) **∃j ∈**{1,...., m}**: f_j _< g_j_

**Definition 7 Pareto set**. Let *F *∈*R ^m ^*be a set of vectors. Then the Pareto set F* of *F *is defined as follows:

F* contains all vectors g ∈ F which are not dominated by any vector f ∈ F, i.e.

(11) F:= {g∈F|∄f∈F: f≻g}

Vectors in F* are called Pareto vectors of F. The set of all Pareto sets of F is denoted as P*(F).

**Definition 8 ϵ-dominance**. Let f, g ∈ *R^m^*. Then f is said to ϵ -dominate g for some ϵ > 0, denoted as f ≻_ϵ _g, iff for all i∈{1,...., m}

(12) $$\left(\text{1}+\epsilon \right)f_{i}\geq g_{i}.$$

**Definition 9 ϵ-approximate Pareto set**. Let F ⊆ *R^m ^*be a set of vectors and ϵ > 0. Then a set *F_ϵ _*is called an ϵ-approximate Pareto set of F, if any vector g ∈ F is ϵ-dominated by at least one vector f ∈ *F_ϵ _*, i.e.

(13) $$\forall \text{g}\in \text{F}:\exists \text{f}\in F_{\epsilon }\text{such that f}{≻}_{\epsilon }\text{g}$$

The set of all ϵ-approximate Pareto sets of F is denoted as P*_ϵ _*(F).

**Definition 10 ϵ-Pareto set**. Let *F *⊆ *R^m ^*be a set of vectors and ϵ > 0. Then a set $F_{\in }^{*}$⊆*F *is called an ϵ-Pareto set of *F *if

(i) $F_{\in }^{*}$ is an ϵ-approximate Pareto set of F, i.e. $F_{\in }^{*}\in P_{\in }\left(F\right)$, and

(ii) $F_{\in }^{*}$ contains Pareto points of X only, i.e. $F_{\in }^{*}\subseteq F^{*}$

The set of all ϵ-Pareto set of F is denoted as $F_{\in }^{*}\left(F\right)$.

### Update of ϵ-Pareto set of the frog population

In order to guarantee the convergence and maintain diversity in the population at the same time, we implement updating of ϵ-Pareto set of the frog population during selection operation [16].

### Fining the global best solution

To order to find the global best solutions, we use the Sigma method [21] to find the best local guide *p_g _*among the population members for the frog *i *of population as follows. In the first step, we assign the value *σ_j _*to each frog *j *in the population. In the second step, *σ_i _*for frog *i *of the population is calculated. Then we calculate the distance between the *σ_i _*and σ_j_, *∀j = 1,...,|A|*. Finally, the frog *k *in the population *A *which its *σ_k _*has the minimum distance to σ_i _is selected as the best local guide for the frog *i*. Therefore, frog p_g _= x_k _is the best local guide for frog *i*. In other words, each frog that has a closer sigma value to the sigma value of the population member, must select that population member as the best local guide. In the case of two dimensional objective space, closer means the difference between the sigma values and in the case of m- dimensional objective space, it means the m-dimensional euclidian distance between the sigma values. The algorithm of the Sigma method can find the best local pg for the frog *i *of the population [21]. Here, the function Sigma calculates the *σ *value and dist computes the euclidian distance. *y_i _*denotes the objective value of the jth element of the population.

### Shuffled frog-leaping algorithm

SFL is a population-based cooperative search metaphor combining the benefits of the genetic-based memetic algorithm and the social behavior based on particle swarm optimization. Shuffled frog leaping algorithm is a new meta-heuristic proposed by Eusuff [31,32,34] for solving problems with discrete decision variables. In the SFL algorithm, a population of randomly generated P solutions forms an initial population *X *= {*x_1_, x_2_,..., x_P_*}, where each solution *x_i _*called a frog is represented by a number of bits *x_i _*= { *x_i1_, x_i2_,..., x_iN _*}.

SFL starts with the whole population partitioned into a number of parallel subsets referred to as memeplexes. Then eachmemeplex is considered as a different culture of frogs and permitted to evolve independently to search the space. Within each memeplex, the individual frogs hold their own ideas, which can be affected by the ideas of other frogs, and experience a memetic evolution. During the evolution, the frogs may change their memes by using the information from the memeplex best *x(b) *or the best individual of entire population *x(g)*. Incremental changes in memotypes correspond to a leaping step size and the new meme corresponds to the frog's new position. In each cycle, only the frog with the worst fitness *x(w) *in the current memeplex is improved by a process similar to PSO. The improving cycle has four steps, in the first step it uses a method which in concept is somehow similar to the discrete particle swarm optimization algorithm, and for the second and third steps it uses the operators of the the binary genetic algorithm (BGA), i.e. mutation and crossover [34].

**Step1**. For *d *= 1,..., *N_bit _*, use Eq.(14) to calculate the speed vector of the worst frog *VW*_*i*_:

(14) $$\begin{array}{c} vw_{id}^{n+1}=\xi (\omega \cdot vw_{id}^{n}+c_{1}\cdot r_{1}\cdot \left(pb_{id}^{n}-xw_{id}^{n}\right)+\\ k\cdot \mu_{1}\cdot c_{2}\cdot r_{2}\cdot \left(gb_{d}^{n}-xw_{id}^{n}\right)+\mu_{2}\cdot c_{3}\cdot r_{3}\cdot \left(xb_{id}-xw_{id}^{n}\right) \end{array}$$

where *i *denotes the worst frog of ith memeplex, *n *represents the iteration number, *Pb_i _*is the best position visited previously by the worst frog of ith memeplex and *XB_i _*is the position of the best frog in ith memeplex, and *ξ *is the constriction factor; c_1_, c_2 _and *c_3 _*are three positive constants called acceleration coefficients (*c_1 _*= *c_2 _*= *c_3 _*= 2); *r_1_, r_2 _*and *r_3 _*are three random numbers uniformly distributed between 0 and 1. *μ_1 _*and *μ_2 _*are called the influence factors, μ_1 _reflects the influence of the global best position on the worst frog and *μ_2 _*reflects the influence of the best position of any memeplex imposed on the worst frog. As a rule *μ_1 _*and *μ_2 _*are positive decimal fractions. The default values of *μ_1 _*and *μ_2 _*are as *μ_1 _*= *μ_2 _*= 0.5. *k *reflects the movement direction, which is selected randomly, thus if *k *= 1 the frog moves towards the global best position, else *k *= -1 and it moves in the opposite direction. *ω *is called the inertia weight, and is calculated from Eq.(14).

The position of the frog is determined using Eq.(15):

(15) $$xw_{id}^{n+1}=boolean\left(xw_{id}^{n}+\text{ v}w_{id}^{n+1}\right)$$

where

$$boolean\left(x\right)=\left\{\begin{array}{c} 1\;\;if\;\;x\geq 0\\ 0\;\;otherwise \end{array}\right)$$

If this process produces a better solution, it replaces the worst frog; otherwise go to the next step.

**Step2**. A mutation operator is applied on the position of the worst frog. In the case of improvement, the resulted position is accepted; otherwise go to the next step.

**Step3**. A crossover operator is applied between the worst frog of the memeplex and the globally best position. The worst frog is replaced if its fitness is improved; otherwise go to the next step.

**Step4**. The worst frog is replaced randomly.

If no improvement becomes possible in this case, then x(w) is replaced by a randomly generated solution within the entire feasible space.

After a predefined number of memetic evolution steps, the frogs in memeplexes are submitted to a shuffling process, where all the memeplexes are combined into a whole population and then the population is again divided into several new memeplexes. The memetic local search and shuffling process are repeated until a given termination condition is reached.

As a predefined number of improvement cycles is reached, memeplexes are shuffled, and if stopping criteria are not met, the algorithm is repeated.

Accordingly, the main parameters of DSFL are: number of frogs P, number ofmemeplexes m, number of processing cycles on each memeplex before shuffling, number of shuffling iterations (or function evaluations), number of bits for any variable, mutation rate, crossover type, the constriction factor, acceleration coefficients and influence factors.

Based on some primary experimental results, the suitable values were found as follows: number of frogs and number of bits for each variable are 60 and 10, respectively, number of processing cycles on each memeplex before shuffling is 10, number of memeplexes is 6. The values of other parameters have been mentioned before. This paper incorporating dynamic population size.

### Dynamic population strategy

Generally, multiple-objective optimization focus on two competing objectives: (1) to quickly converge to the true Pareto front and (2) to maintain the diversity of the solutions along the resulting Pareto front. Because maintaining the diversity will slow down the convergence speed and may degrade the quality of the resulting Pareto front, these two objectives are in conflict each other. In this paper, we adopt dynamically adjusting the population size to explore the search space in balance between two competing objectives.

### Initializing the population

The initial population is get by running state-of-art MOEA (NSGA-II [37]) with 50 individuals and 20 generations to produce the initial population of MODPSFLB.

### Adding population size

Population adding strategy mainly consist in increasing the population size to ensure sufficient number of individuals to contribute to the search process and to place those new individuals in unexplored areas to discover new possible solutions. Based on the strategies of dynamic population size [29], the procedures proposed in literature [38] is proposed to facilitate exploration and exploitation capabilities for MODPSFLB.

### Decreasing population size

To prevent the excessive growth in population, a population decreasing strategy [27] is used to adjust the population size. Sigma value is utilized to select potential frogs to be deleted. After computing all the distance between Sigma value of each frog and Sigma value of its corresponding best local guide, the rank of the distance of each frog can be attained. If the removal of frogs is only based upon the distance rank of each frog, then there is a possibility of eliminating an excessively large quantity of frogs in which some may carry unique schema to contribute in the search process. A selection ratio is implemented to regulate the number of frogs to be removed and to provide some degrees of diversity preservation at the same time. A selection ratio inspired by Coello and Montes [39] is used to stochastically allocate a small percentage of frogs in the population for removal.

### MODPSFLB biclustering algorithm

We incorporates dynamic population strategy into multi-objective shuffled frog leaping biclustering (MOSFLB) [38] algorithm, and propose a multi-objective dynamic population shuffled frog-leaping biclustering (MODPSFLB) to mine biclusters from the microarray datasets to attain the global optimum solutions. The proposed algorithm consist of the following three strategies: (1) ϵ-dominance to quicken convergence speed; (2) Sigma method to find good local guides; (3) population-growing strategy to increase the population size to promote exploration capability; and (4) population declining strategy to prevent the population size from growing excessively. The pseudo-code of the proposed MODPSFLB algorithm is given in Algorithm 1.

**Algorithm 1**: MODPSFLB Algorithm

**Input**: microarray data, minimal *MSR *δ, α

**Output**: the best solutions, that is, the found biclusters

Begin

Initialize the frog population A according to the population initializing stragery

**While **not terminated **do**

Calculate fitness for each frog

Add the size of population A according to the population adding stragery

Divide the population into several memeplexes

**For **each memeplex

Determine the best and worst frogs

Improve the worst frog position x(w) using Eq.(15)

**If **no improvement in this case **then**

x(w) is replaced by a randomly generated frog within the entire feasible space

End for

Combine the evolved memeplexes

Select the best frogs using Sigma method and ϵ-dominance

Decrease the size of population A according to the population decreasing stragery

End while

**Return ***At *the set of biclusters

END

MODPSFLB algorithm iteratively updates the frogs population until maximum number of generation are reached and converge to the optimal solution set.

## Results

Mitra and Banka applied MOEA to solve biclustering problem and proposed MOE Biclustering (MOEB) [17]. To obtain the diversity of optimal solution, we apply the proposed MODPSFLB algorithm to mine biclusters from two well known datasets and compare the diversity and convergence of the algorithm with MOEB, MOPSOB [40] and MOSFLB algorithm. The biological significance of the biclusters found by MODPSFLB is given in the end.

### Datasets and data preprocessing

The first dataset is the yeast Saccharomyces cerevisiae cell cycle expression data [41], and the second dataset is the human B-cells expression data [42].

The yeast dataset collects expression level of 2,884 genes under 17 conditions. All entries are integers lying in the range of 0-600. Out of the yeast dataset there are 34 missing values. The 34 missing values are replaced by random number between 0 and 800 [5].

The human B-cells expression dataset is collection of 4,026 genes and 96 conditions, with 12.3% missing values, lying in the range of integers -750-650. The missing values are replaced by random numbers between -800-800^[5]^. However, those random values affect the discovery of biclusters [43]. The parameter δ, for the yeast data is set δ = 300, for the human B-cells expression data δ = 1200.

### Experiments

MODPSFLB algorithm is implemented in JAVA programming language and is performed on a 1.7 GHz Pentium 4 PC with 512 M of RAM running Windows XP. To evaluate its performance, the proposed algorithm is compared to MOEB, MOPSOB [40] and MOSFLB algorithm on two well known datasets [41,42].

### Yeast dataset

In Table 1, the information of ten biclusters out of the one hundred biclusters found on the yeast dataset are shown. Table 1 shows that the first hundred biclusters found by the proposed MOSFLB algorithm cover 77.7% of the genes, 100% of the conditions and in total 57.2% cells of the expression matrix. The biclusters found by MOSFLB algorithm cover 76.7% of the genes, 100% of the conditions and in total 54.3% cells of the expression matrix. The biclusters found by MOPSOB [40] cover 73.1% of the genes, 100% of the conditions and in total 52.4% cells of the expression matrix. While an average coverage of 51.34% cells is reported in MOEB [17].

**Table 1:** Information of biclusters found on yeast dataset

Bicluster	Genes	Conditions	Residue	Row variance
1	101	15	215.62	749.17
6	514	10	289.65	955.25
14	858	10	322.58	702.36
22	478	11	298.68	885.64
31	123	12	201.88	699.87
36	801	8	221.88	687.18
44	1125	13	236.47	598.68
56	847	11	208.48	748.54
75	546	9	250.14	664.13
89	89	17	210.88	666.57

Table 1 shows the number of genes and conditions, the mean squared residue and the row variance of ten biclusters out of the one hundred biclusters found on the yeast dataset.

**Figure 2:**
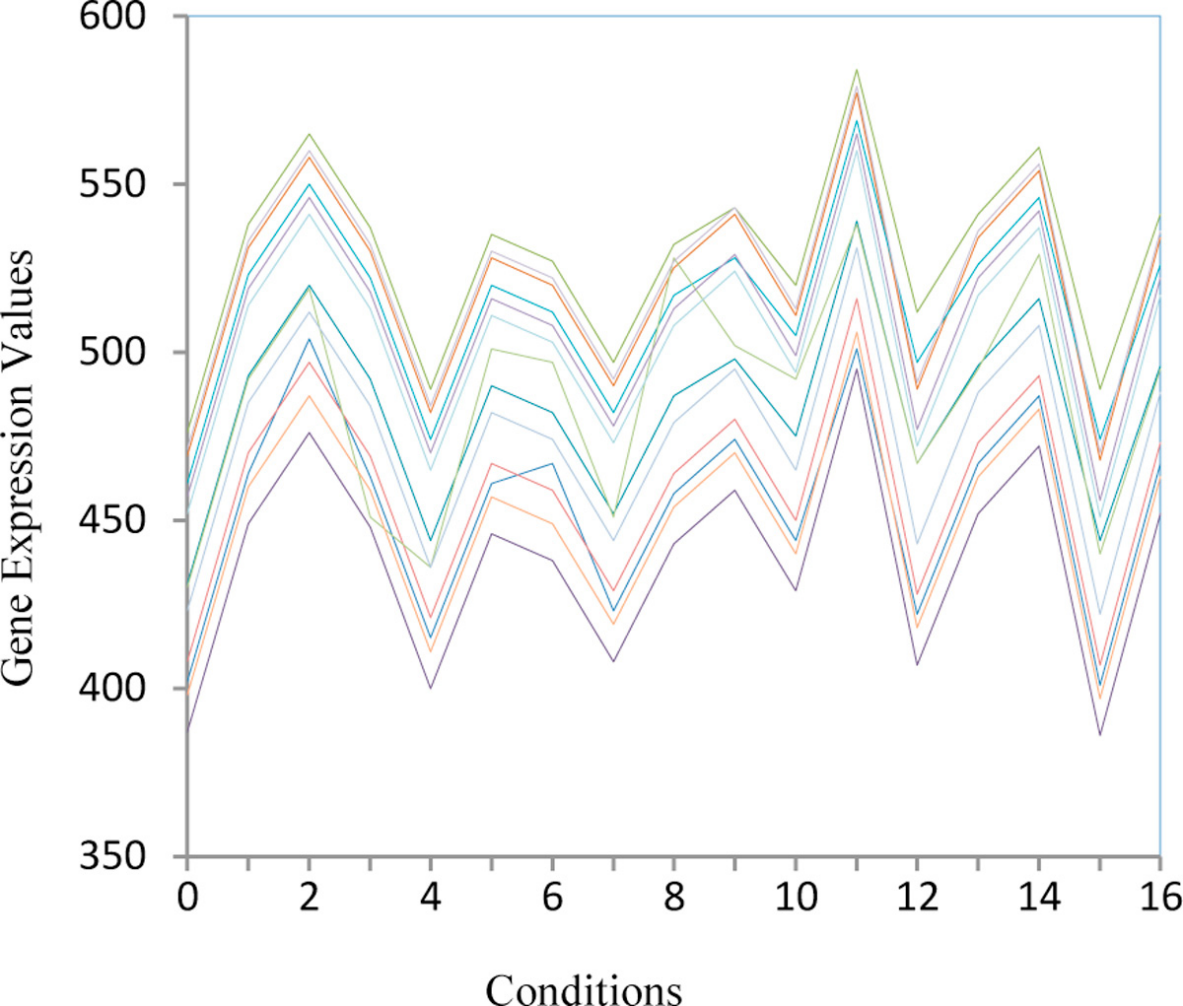
**Small biclusters of size 24 × 17 on the yeast dataset**. Figure 2 shows the expression value of 24 genes under 17 conditions from the small biclusters (bicluster 63).

Figure 2 depicts sample gene expression profiles for small biclusters (bicluster 63) for the yeast dataset. They show that 24 genes present a very similar behaviour under 17 conditions.

### Human B-cells expression dataset

Table 2 shows the information of ten biclusters out of the one hundred found on the human dataset. From Table 2, we know that the first hundred biclusters found by the proposed MOSFLB algorithm cover 42.1% cells of microarray dataset (53% of the genes and 100% of the conditions). However, the one hundred biclusters found by MOSFLB algorithm cover 40.8% cells of microarray dataset (51.2% of the genes and 100% of the conditions). The one hundred biclusters found by MOPSOB [40] on the human dataset cover 35.7% cells of dataset (46.7% of the genes and 100% of the conditions), whereas an average of 20.96% cells are covered in MOEB [17].

**Table 2:** Biclusters found on human dataset

Bicluster	Genes	Conditions	Residue	Row variance
1	882	34	987.54	3587.26
4	666	54	1087.25	4201.36
11	1024	36	773.69	2930.64
17	1102	39	1204.65	3698.84
24	968	37	1110.25	3548.45
35	805	41	844.44	2987.01
39	871	48	2874.17	2140.36
44	1208	29	885.74	3587.45
59	258	86	777.58	2874.94
88	1508	59	1405	6658.45

Table 2 shows the number of genes and conditions, the mean squared residue and the row variance of ten biclusters out of the one hundred biclusters found on the human dataset.

### Comparative analysis

We compare the proposed MODPSFLB algorithm with MOPSOB, MOSFLB and DMOPSOB algorithm on the yeast dataset and the human dataset and the results are showed in Table 3.

**Table 3:** Comparative study of three algorithms

	MOPSOB		MOSFLB		DMOPSOB		MODPSFLB	
**Dataset**	**Yeast**	**Human**	**Yeast**	**Human**	**Yeast**	**Human**	**Yeast**	**Human**

Avg. MSR	218.54	927.47	215.98	913.53	216.13	905.23	212.8	904.9
Avg. size	10510.8	34012.24	1109.23	35507.22	11213.5	35442.98	11220.7	35601.8
Avg. genes	1102.84	902.41	1148.21	928.12	1151.25	932.57	1154.21	933.9
Avg. conditions	9.31	40.12	9.78	43.11	9.59	42.78	9.81	43029
Max size	15613	37666	15709	37871	14770	37231	14827	37486
Avg. time	120.78	328.56	111.41	319.88	100.47	310.34	88.24	287.98

Table 3 compares the performance of two algorithms. It gives the average of mean squared residue and the average size of the found biclusters, and gives computation cost of two algorithms.

From Table 3, the biclusters found by MODPSFLB has a slightly higher squared residue and a higher bicluster size than those by the other three algorithm on both yeast dataset and human dataset. It is clear from the above results that the proposed MODPSFLB algorithm performs best in maintaining the diversity of solutions.

As for the computation cost, Table 3 shows that the computation time of MODPSFLB is least, that is 88.24s on yeast dataset and 287.98s on human dataset, is superior to that of the other thress algorithms. From Table 3, we alse know that the algorithm adopting dynamic population strategy has less the computation cost than the algorithm not adopting dynamic population strategy. This show that dynamic population strategy can quicken optimization process.

In total it is clear from the above results that the proposed MODPSFLB algorithm performs best in maintaining diversity, achieving convergence.

### Biological analysis of biclusters

We determine the biological relevance of the biclusters found by MODPSFLB on the yeast dataset in terms of the statistically significant GO annotation database. The gene ontology (GO) project (http://www.geneontology.org) provides three structured, controlled vocabularies that describe gene products in terms of their associated biological processes, cellular components and molecular functions in a species-independent manner. To better understand the mining results, we feed genes in each bicluster to Onto-Express (http://vortex.cs.wayne.edu/Projects.html) and obtain a hierarchy of functional annotations in terms of Gene Ontology for each bicluster.

The degree of enrichment is measured by p-values which use a cumulative hyper geometric distribution to compute the probability of observing the number of genes from a particular GO category (function, process and component) within each bicluster. For example, the probability *p *for finding at least *k *genes from a particular category within a bicluster of size n is given in Eq.(16).

(16) $$p=1-\overset{k-1}{\underset{i=0}{\sum }}\frac{\left(\begin{array}{c} m\\ i \end{array}\right)\left(\begin{array}{c} g-m\\ n-i \end{array}\right)}{\left(\begin{array}{c} g\\ n \end{array}\right)}$$

Where m is the total number of genes within a category and g is the total number of genes within the genome. The p-values are calculated for each functional category in each bicluster to denote how well those genes match with the corresponding GO category.

Table 4 lists the significant shared GO terms (or parent of GO terms) used to describe the set of genes in each bicluster for the process, function and component ontologies. Only the most significant common terms are shown. For example for cluster C_1_, we find that the genes are mainly involved in Oxidoreductase activity. The tuple (n = 13, p = 0.00051) means that out of 101 genes in cluster C_1_, 13 genes belong to Oxidoreductase activity Function, and the statistical significance is given by the p-value of 0.00051. Those results mean that the proposed MODPSFLB biclustering approach can find biologically meaningful clusters.

**Table 4:** Significant GO terms of genes in three biclusters

**Cluster No**.	No. of genes	Process	Function	Component
1	101	Lipid transport (n = 21, p = 0.00389)	Oxidoreductase activity (n = 13, p = 0.00051)	Membrane (n = 12, p = 0.0023)

12	71	Physiological process (n = 43, p = 0.0043)	MAP kinase activity (n = 7, p = 0.00126)	Cell (n = 32, p = 0.00194)

33	58	Protein biosynthesis (n = 27, p = 0.00216)	Structural constituent of ribosome (n = 17, p = 0.00132)	Cytosolic ribosome (n = 11, p = 0.00219)

Table 4 lists the significant shared GO terms which are used to describe genes in each bicluster for the process, function and component ontology.

## Conclusions

This paper proposes a novel multi-objective dynamic population shuffled frog-leaping biclustering framework for mining biclusters from microarray datasets. We focus on finding maximum biclusters with lower mean squared residue and higher row variance. Those three objective are incorporated into the framework with three fitness functions. We apply the following techniques: a SFL method to balance and control the search process, population adding method to dynamically grows new individuals with enhanced exploration and exploitation capabilities, population decreasing strategy to balance and control the dynamic population size, and final to quicken convergence of the algorithm.

The comparative study of MODPSFLB and three state-of-the-art biclustering algorithms on the yeast microarray dataset and the human B-cells expression dataset clearly verifies that MODPSFLB can effectively find significant palocalized structures related to sets of genes that show consistent expression patterns across subsets of experimental conditions. The mined patterns present a significant biological relevance in terms of related biological processes, components and molecular functions in a species-independent manner.

## Competing interests

The authors declare that they have no competing interests.

## Authors' contributions

JL was primarily responsible for the design of MODMSFLB to mine biclusters from gene expression data and drafted the manuscript. ZL and XH were involved in study design and coordination and revised the manuscript. YC and FL conducted the algorithm design.
